# Land‐Use Effects on Surface Water Quality in Temperate Lowland Peatlands

**DOI:** 10.1111/gcb.70988

**Published:** 2026-07-05

**Authors:** Teresa Silverthorn, Dan Aberg, Francesca Baker, Chris Bell, Chris D. Evans, Caroline Gurd, Ian Holman, Graham Howell, Angus McEwen, Ross Morrison, Josh Oakley, Francesca Southon, Mike Peacock

**Affiliations:** ^1^ Department of Geography and Planning, School of Environmental Sciences University of Liverpool Liverpool UK; ^2^ School of Environmental Science Simon Fraser University Burnaby Canada; ^3^ School of Environmental and Natural Sciences Bangor University Bangor UK; ^4^ UK Centre for Ecology & Hydrology Bangor UK; ^5^ School of Environment, Earth and Ecosystem Sciences The Open University Milton Keynes UK; ^6^ Cranfield Water Science Institute Cranfield University Cranfield UK; ^7^ UK Centre for Ecology & Hydrology Wallingford UK; ^8^ Department of Aquatic Sciences and Assessment Swedish University of Agricultural Sciences Uppsala Sweden

**Keywords:** biogeochemistry, greenhouse gases, land use, lowland peatlands, macronutrients, nutrient stoichiometry, peatland drainage, peatland rewetting, water chemistry

## Abstract

Peatlands are important ecosystems for carbon storage, water regulation, water quality and other valuable ecosystem services that have been heavily impacted by drainage and conversion to grassland and cropland. Rewetting, or raising of the water table closer to the ground surface, is proposed as a solution to reduce peatland subsidence and carbon losses. Both peatland drainage and peatland rewetting may have unintended impacts on water quality, either by introducing solutes from the water used for rewetting, or by leaching of substances accumulated in peat. Here, we examined the effects of land use on UK lowland peat surface water chemistry by collecting a total of 350 surface water samples from 77 individual water bodies (ditches, streams, ponds) in 2024, across 33 sites representing the range of temperate lowland peatland types. Samples were measured for a wide array of chemical determinands including: carbon (C), nitrogen (N) and phosphorus (P), heavy metals, other anions and cations, and dissolved greenhouse gas concentrations. We found some clear patterns related to land use, notably high nitrate concentrations in surface waters draining croplands. Macronutrient stoichiometry revealed similar patterns at grassland, conservation‐managed and rewetted sites indicative of N and P co‐limitation, suggesting that rewetting leads to a nutrient balance comparable to natural conditions. Peatland type (bog vs. fen) was more important than land use in explaining patterns in most ion concentrations. Site and regional histories were more important than land use for patterns of heavy metal concentrations, indicating the importance of considering regional historical industrial activity. Overall, peatland land use can be an important driver of key nutrients and their stoichiometry, with no consistent negative impacts of peatland rewetting on water quality nor greenhouse gases.

## Graphical abstract

We examined the effects of land use on UK lowland peat surface water chemistry, finding similar macronutrient stoichiometry at conservation‐managed and rewetted sites (indicative of nitrogen and phosphorus co‐limitation), suggesting that rewetting leads to a nutrient balance comparable to natural conditions.

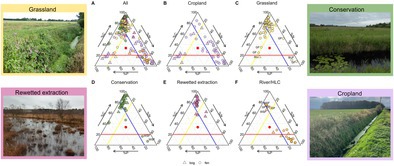

## 1 Introduction

Peatlands are important ecosystems for carbon storage, water regulation, water quality, along with other valuable ecosystem services (Page and Baird 2016). Despite covering only 3% of global land area, peatlands store about 600 petagrams (Pg) of carbon (Yu et al. 2010)—nearly double the global living forest biomass (Pan et al. 2011). Yet, around the globe, peatlands have been drained and degraded, resulting in an estimated ~20% loss of wetlands globally, with drainage for agriculture as the leading cause of this loss (Fluet‐Chouinard et al. 2023). Peatland drainage and subsequent aeration, although improving the soil productivity, also promote microbial respiration, thereby accelerating organic matter decomposition (Page and Baird 2016). As a result, drained peatlands release 2 Pg of carbon dioxide equivalent emissions annually (excluding fire), which accounts for 4% of global anthropogenic emissions (United Nations Environment Programme [UNEP] 2022).

In additional to enhancing greenhouse gas (GHG) emissions, peatland drainage also contributes to land surface subsidence. Peat is ~90% water by volume when saturated and, as such, drainage of previously saturated soils can result in a loss of buoyancy, compaction and therefore a reduction of peat volume. In combination with peat loss resulting from accelerated microbial decomposition, this leads to a lowering of the ground surface, that is, peat subsidence (Page et al. 2020). In some intensively drained areas of north‐west Europe, 2–5 m of peat have been lost since the mid‐1800s, putting some areas below sea level (Hutchinson 1980; Leifeld et al. 2011). The remaining “lifetimes” of some agricultural lowland peats are now less than one century (Evans, Morrison, et al. 2016). Given these pressing challenges, strategies to mitigate peat degradation and GHG emissions are rapidly needed.

The widespread drainage and land use conversion of peatlands, which turns peatlands from net carbon sinks to net sources of GHG emissions, highlights the challenging balance between food security and climate change mitigation (Freeman et al. 2022). In response to these concerns, governments around the world are mandating large‐scale peatland rewetting activities, sometimes accompanied by restoration or by more sustainable agricultural practices such as paludiculture (wetter farming) (Bockermann et al. 2025; Freeman et al. 2022; Mulholland et al. 2020). For example, European Union countries must rewet 25% of drained peatlands by 2030 (Regulation (EU) 2024/1991). Peatlands in the UK are among some of the most heavily impacted worldwide, alongside regions in Southeast Asia, China, the United States, and Europe (Fluet‐Chouinard et al. 2023). It is estimated that about 90% of UK lowland peat has been drained for agriculture (Evans et al. 2017). As such, UK lowland peatlands represent a globally significant example of extensive and long‐standing modification, providing valuable insights to regions at earlier stages of degradation as well as generally informing global peatland management and restoration efforts.

In contrast to many other temperate regions, the majority of the UK's peatland area is covered by blanket bogs, a peat type that occurs in hilly landscapes in high‐rainfall oceanic regions, and therefore has a limited global extent (Gallego‐Sala and Prentice 2013). In contrast, lowland peatlands (comprising lowland fens and lowland raised bogs) occupy only ~15% of the UK peatland area (Page et al. 2020), but likely the majority of peatland area globally, occurring in both coastal and continental locations (Greifswald Mire Centre 2022). Lowland peatlands in the UK are subject to disproportionately high levels of land‐use pressure due to their high fertility, equable climate and accessibility, often in proximity to population centres (Haddaway et al. 2014; JNCC 2011). Given their contrasting hydrological and biogeochemical properties, and historic and current land‐use, we expect these systems to have different water quality characteristics when compared to blanket bogs.

To date, most studies examining the effects rewetting have focused on GHG emissions (Evans et al. 2021; Freeman et al. 2022) and on fluvial organic carbon (Evans et al. 2006). However, peatland drainage can lead to an accumulation of substances within the peat profile from agricultural and/or industrial sources (Novak and Pacherova 2008). Raising the water table may mobilize these accumulated substances, leading to the leaching of heavy metals (Rothwell et al. 2010) and nutrients (Daniels et al. 2012), as well as the transport of suspended sediments (Evans et al. 2006) into receiving surface waters. Although the extent of this will depend on a variety of factors such as peatland type, historic and current land‐use intensity, the degree of rewetting, etc. (Zak et al. 2010; Zak and Gelbrecht 2007). When rewetting former agricultural lands, the mobilization of phosphate (PO_4_^3−^) and ammonium (NH_4_^+^) which have accumulated in the top peat layers from fertiliser application is a particular concern (Zak and Gelbrecht 2007). Sulphate (SO_4_^2−^) and potassium (K) have also been observed to increase after rewetting when the concentrations are high in rewetting source waters (Koerselman et al. 1993; van der Laan et al. 2024). Any post‐rewetting leaching of substances may have implications for aquatic ecology, drinking water, GHG emissions and recreational water use (Breen et al. 2018; Jones 1992; Vuori 1995; Xu et al. 2018). In contrast, intact, accumulating peatlands can store nutrients and persistent pollutants, preventing their leaching into surface waters (Martin‐Ortega et al. 2014). Consequently, rewetting may have a positive effect, where substances present in polluted water used for rewetting become bound to the peat matrix (Berset et al. 2001; Rawlins et al. 2012), thereby improving downstream water quality—delivering co‐benefits for GHG mitigation and water quality improvement through rewetting. Here, we present one of the first national‐scale assessments of an extensive range of water quality indicators in relation to peatland drainage, rewetting, and land use effects.

Our objective was to examine the effects of current and past land use on UK lowland peat surface water quality. We were also interested in examining the effects other relevant factors such as site/regional history and peatland type (bog vs. fen). In addition to water chemistry, we measured dissolved GHG concentrations at a subset of sites, to explore their linkages with water quality. We collected water samples from waterbodies (ditches, ponds, small lakes) at conservation‐managed sites (i.e., with vegetation typical of intact peatlands), rewetted peat extraction sites, as well as ditches at drained sites currently under active grassland or cropland use. Additionally, we took samples from watercourses that are typically used as water sources for cropland irrigation and for rewetting.

## 2 Methods

### 2.1 Field Sampling

#### 2.1.1 Water Quality

**FIGURE 1:**
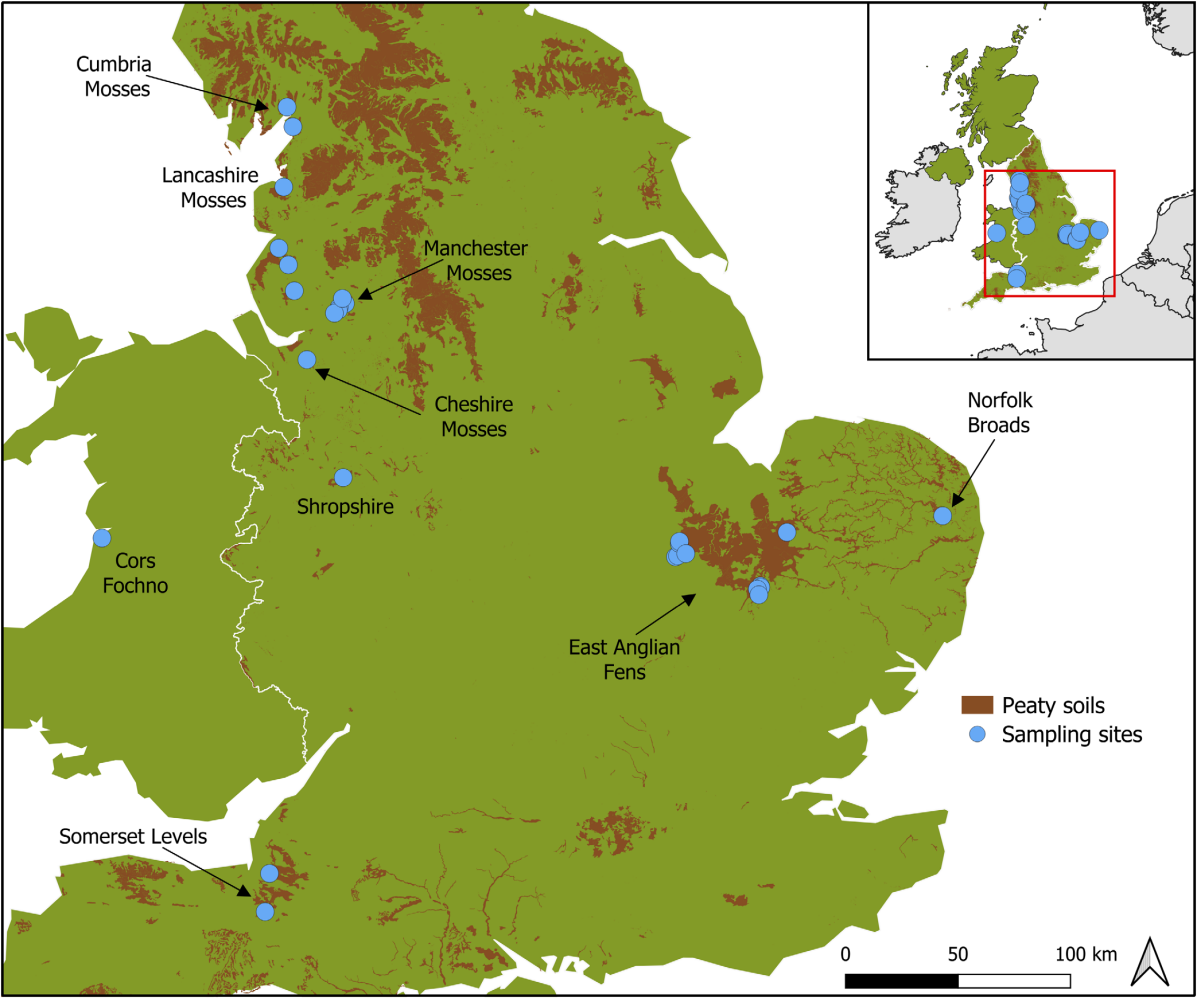
Sampling sites for lowland peat surface water quality across England and Wales. England peaty soils data from Natural England, British Geological Survey, Cranfield University (NSRI), Ordnance Survey and partners. Inset map shows the UK with a red box outlining the main map extent.

We collected water samples from 350 unique sample points, from 77 individual water bodies, at 33 sites across representative lowland peatlands in England and Wales (Figure 1, Table S1). Sites are temperate but varied in climate conditions, particularly for rainfall, with mean annual temperature (MAT) ranging from 8.5°C (north‐west England) to 10.8°C (eastern England) and mean annual precipitation (MAP) ranging from ~570 mm (eastern England) to ~1400 mm (north‐west England) (UK Met Office 2025). Most sites (*n* = 22) were sampled repeatedly on at least three and up to six occasions, while a smaller number of sites were sampled only once (*n* = 10) or twice (*n* = 1). Sites were sampled between April 26 and November 22, 2024. Sampled sites included both bogs (ombrotrophic peatlands that receive water and nutrients almost exclusively from atmospheric deposition) and fens (minerotrophic peatlands fed by groundwater or surface water higher in dissolved ions and nutrients) (Bleuten et al. 2006). Peatland type was confirmed by the Lowland Peat Soil Survey of England and Wales (Burton and Hodgson 1987) and the Landis Soilscapes dataset (Cranfield University 2025). Sampled waterbodies drained peatlands under a range of management conditions, which will be referred to as land use throughout: cropland, grassland, rewetted (former) peat extraction, and conservation‐managed. The land use classifications were determined through expert site knowledge confirmed by field observations and discussions with landowners. The majority of measurements were from ditch surface waters, but surface waters from ponds (six measurements) and lakes (seven measurements) were also sampled. Where feasible, we also collected water samples from rivers and high‐level carriers (HLCs) for reference. HLCs are any watercourse, often a channel, that is elevated relative to the ground surface to convey water without mixing with the local catchment (Anglian Water 2024). These rivers and HLCs are frequently used as water sources for crop irrigation, raising water tables, and full rewetting; thus, it is useful to know the chemical composition of their waters.

We collected water samples in 125 mL polypropylene bottles pre‐rinsed in the field with sample water. After collection, samples were stored in the dark at 4°C. Samples were then sent to the Ecosystems Laboratories at the School of Environment, Earth and Ecosystem Sciences, Open University, UK, for analysis. Upon arrival, samples were either placed in cold storage (4°C) in the dark for several days before being analysed, or frozen immediately to preserve them for analysis weeks/months later. In late September and early October 2024, we additionally collected a total of 60 samples at a subset of 20 sites for analysis of dissolved mercury (Hg) using UKAS‐accredited (ISO/IEC 17025:2017) National Laboratory Service (NLS) protocols. Sampled were filtered in the field using 0.45 μm syringe filters and injected into 100 mL Pyrex bottles containing 4 mL of 17% hydrochloric acid, before being transported to the NLS in temperature‐controlled boxes.

#### 2.1.2 Greenhouse Gases

We measured surface water GHG concentrations (*n* = 64 samples) at 59 waterbodies at a subset of sites (*n* = 18) on one to two occasions from May 16 to July 9, 2024, as a supplement to the water quality data. We acknowledge that GHG concentrations may be influenced by the weather and antecedent conditions, but we hypothesize that the influence of land use will be the dominant signal. GHG concentrations were measured using the headspace equilibration technique (Hope et al. 1995). Briefly, 30 mL of surface water was collected in a 60 mL plastic syringe and equilibrated with 30 mL of ambient air by shaking vigorously for 1 min. This equilibrated headspace gas was then injected into pre‐evacuated 12 mL glass exetainers (Labco Ltd., Lampeter, Wales) and analysed for CO_2_, CH_4_, and N_2_O concentrations on a gas chromatograph (Varian 450 GC, Agilent Technologies, CA, USA). The GHG concentrations were corrected for dilution and converted to dissolved concentrations according to solubility coefficients (Weiss 1974; Weiss and Price 1980; Wiesenburg and Guinasso 1979), considering the water temperature, elevation, and barometric pressure at each site. We estimated GHG emissions using the dissolved gas concentrations and a ditch‐specific gas transfer velocity of 0.33 m d^−1^, which is typical of lowland peat ditches in the UK (Peacock et al. 2017).

### 2.2 Laboratory Analyses

Apart from Hg analysis, all water chemistry variables were measured at the Ecosystems Laboratories, Open University, UK. Dissolved Hg concentrations were measured via cold vapour atomic fluorescence spectroscopy at the NLS. Electrical conductivity (EC) and pH were measured on unfiltered samples using an Orion VERSA STAR multiparameter meter (Thermo Scientific, USA). Dissolved organic carbon (DOC) was measured either by difference (total carbon [TC]—inorganic carbon [IC]) on an Elementar Vario TOC Cube elemental analyser (Elementar Analysensysteme GmbH, Langenselbold, Germany) or as Non‐Purgeable Organic Carbon on a total organic carbon (TOC) analyser (Shimadzu TOC‐L, Shimadzu, Japan). Inductively Coupled Plasma Optical Emission Spectroscopy (ICP‐OES) and SPS 4 autosampler (Agilent Technologies, USA) were used to measure concentrations (on unfiltered samples) of elements: total phosphorous (TP), iron (Fe), manganese (Mn), aluminium (Al), copper (Cu), lead (Pb), arsenic (As), cadmium (Cd), chromium (Cr), nickel (Ni), silicon (Si), and zinc (Zn). Anions fluoride (F^−^), chloride (Cl^−^), nitrate (NO_3_^−^), nitrite (NO_2_^−^), phosphate (PO_4_^3−^), and sulphate (SO_4_^2−^) as well as cations ammonium (NH_4_^+^), sodium (Na), potassium (K), calcium (Ca), magnesium (Mg), and lithium (Li) were analysed using ion chromatography on filtered samples (0.45 μm) (930 Compact IC Flex and 889 IC Sample centre, Metrohm, Switzerland). Both ion chromatography and ICP analyses were run on pure undiluted samples; the ion chromatography runs were diluted with 18 MΩ·cm water when necessary, and the ICP with 2% nitric acid. For more details on these analyses, including the determination of the lower limit of detection (LLD) and limit of quantification (LoQ) for each determinand, see the Text S1.

### 2.3 Statistical Analyses

All statistical analyses were conducted using the programming software R, version 4.5.0 (R Core Team 2025), using base R functions unless otherwise specified. Concentrations below the LLD were treated as LLD*0.5, in line with best practices used in other similar studies (Finckh et al. 2022; Holman et al. 2010). To evaluate the effects of land use and peatland type on the water quality variables we used linear mixed‐effects models (LMMs), modelling each variable separately using the *lmer* function from the lme4 package (Bates et al. 2015). We included sampling point nested within site as a random effect to account for repeated measures. Given our focus on land use effects, we do not conduct an in‐depth analysis of the temporal variability for each determinand, although we provide time series plots of key nutrients in the Supporting Information (Figure S1). To evaluate the effects of land use on GHG concentrations and emissions we used linear models (LMs), modelling each variable separately using the *lm* function. We examined model residuals and Q‐Q plots to assess homoscedasticity and normality. In some cases, we transformed the response variable and removed outliers to improve model fit. For the LMMs, the fixed effects were tested using Type III Analysis of Variance (ANOVA) using Satterthwaite's approximation for degrees of freedom using *anova* from the lmerTest package (Kuznetsova et al. 2017), or base R *anova* for the LMs. Pairwise comparisons between land use categories were performed using estimated marginal means using the emmeans package (Lenth 2025). We used Spearman (r_S_) correlations to test for monotonic relationships between variables, displayed as a correlation matrix using the corrplot package (Wei and Simko 2024). In all cases, significance was accepted at *p* < 0.05. Means are given ± one standard deviation (SD) throughout.

To compare nutrient stoichiometry between land use types, we created C:N:P ternary plots using the ggtern package (Hamilton and Ferry 2018) following methods adapted from Smith et al. (2017) and Jarvie et al. (2018). Total inorganic N (TIN) was calculated as the sum of NH_4_^+^‐N, NO_2_^−^‐N, and NO_3_^−^‐N. PO_4_^3−^‐P was used as a proxy for reactive P as we had more data points than for TP (which had a strong linear relationship with PO_4_^3−^‐P: *R* ^2^ = 0.93, *p* < 0.0001). We used DOC to represent C on the ternary plot as we did not measure biologically available OC. As such, we may over‐estimate the pool of C accessible to microbial communities; therefore, interpretations of N and/or P limitation should be treated as suggestive rather than definitive. After converting all concentrations to molar units (μmol L^−1^), TIN concentrations and P concentrations were multiplied by 6.625 and 106, respectively to place the Redfield Ratio (C:N:P 106:16:1) in the centre of the ternary plot. In theory, this central zone represents “optimal” ratios of C:N:P for uptake by aquatic algae where nutrient concentrations exceed limiting concentrations (Smith et al. 2017). Sampling points falling below the 20% threshold are referred to as C, N, or P “limited”, but note that this is *potential* nutrient limitation as only partial nutrient pools and not exclusively biologically available pools were measured (Jarvie et al. 2018; Smith et al. 2017). Moreover, stoichiometric concentrations are relative, not absolute; for example, a low N:P may not indicate N limitation if absolute N concentrations are high (Bothwell 1985).

## 3 Results

### 3.1 Carbon

**FIGURE 2:**
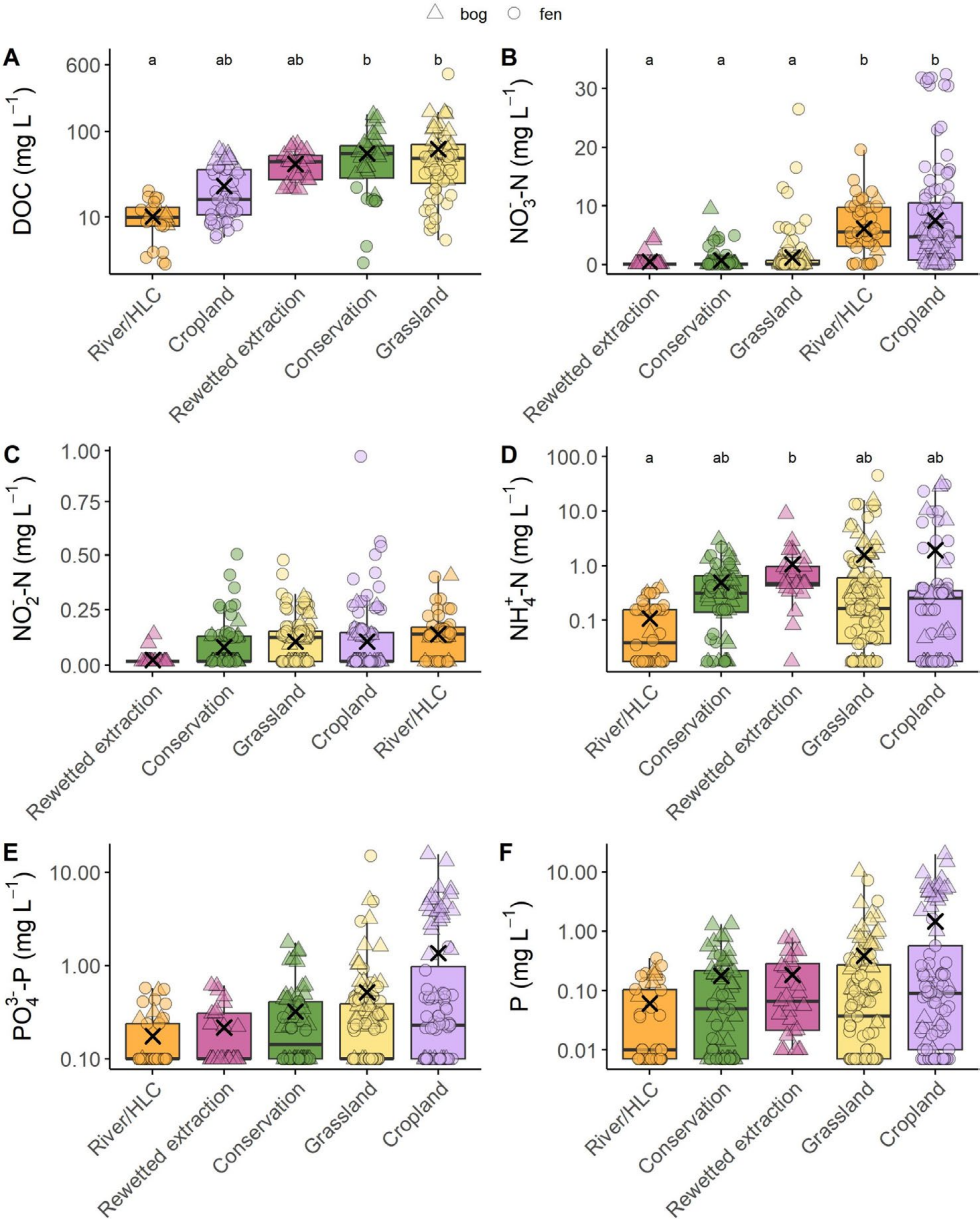
Key nutrient concentrations in lowland peat surface waters by land use type, ordered by increasing mean for each determinand. Note that some variables are plotted on a logarithmic scale for clarity. Box plots display the median (horizontal line), mean (“X”), 25th and 75th percentiles, and whiskers display values 1.5× interquartile range. Bars sharing the same lowercase letters (or with no letters) are not significantly different (pairwise Tukey adjusted, *p* < 0.05).

Land use had a significant effect on DOC (*p* = 0.003; Table 1, Figure 2A), with the highest mean concentrations at the grassland sites (62.5 mg L^−1^ ± 70.0) and lowest at the river/HLC sites (10.0 ± 5.3). Pairwise comparisons showed significantly higher DOC at the grassland sites than river/HLCs (*p* = 0.005). DOC concentrations were two times higher at the bog (57.6 ± 36.2) than fen (27.1 ± 52.0) sites (*p* < 0.0001; Table S2).

**TABLE 1:** Type III ANOVA results from LMMs assessing land use effects on water quality variables. For each variable the degrees of freedom (numerator and denominator), *F* value, and *p* value are noted. In most cases the response variables were log transformed (except for pH; Cr was cube root transformed; F^−^ was square root transformed). In some cases, outliers were removed: *n* = 1 for EC, DOC, Cd, and Zn; *n* = 2 for Mn; and *n* = 3 for Fe. Significant effects (*p* < 0.05) are bolded.

Variable	df (num)	df (den)	*F* value	*p* value
pH	**4**	**51.80**	**3.34**	**0.02**
EC	**4**	**50.98**	**3.63**	**0.01**
DOC	**4**	**40.10**	**4.76**	**0.003**
NO_3_^−^‐N	**4**	**36.77**	**17.03**	**< 0.0001**
NH_4_^+^‐N	**4**	**47.71**	**2.75**	**0.04**
NO_2_^−^‐N	4	47.97	2.36	0.07
PO_4_^3−^‐P	4	56.75	0.66	0.62
P	4	89.92	1.87	0.12
As	4	44.67	0.09	0.99
Cd	4	76.61	0.66	0.62
Cr	4	32.14	0.86	0.50
Cu	4	83.86	0.13	0.97
Fe	**4**	**58.06**	**2.77**	**0.04**
Mn	**4**	**47.50**	**2.88**	**0.03**
Ni	**4**	**52.67**	**5.41**	**0.001**
Pb	4	44.35	1.04	0.40
Zn	4	53.82	1.82	0.14
Al	4	57.20	1.76	0.15
Ca	**4**	**50.31**	**4.75**	**0.003**
Cl^−^	4	56.82	1.51	0.21
F^−^	4	54.71	2.27	0.07
K	4	47.86	1.81	0.14
Li	4	27.96	0.68	0.61
Mg	**4**	**54.66**	**4.54**	**0.003**
Na	4	55.22	1.40	0.25
Si	4	58.47	1.10	0.36
SO_4_^2−^‐S	**4**	**52.55**	**6.56**	**0.0002**

### 3.2 Nitrogen

Land use had a significant effect on NO_3_^−^‐N (*p* < 0.0001) and NH_4_^+^‐N concentrations (*p* = 0.04), but not on NO_2_^−^‐N (*p* = 0.07) (Table 1, Figure 2B–D). The highest mean NO_3_^−^‐N concentrations were observed at the cropland sites (7.5 mg L^−1^ ± 9.0) and lowest at the rewetted extraction sites (0.51 ± 1.2). Pairwise comparisons showed significantly higher NO_3_^−^‐N at the river/HLC and cropland sites compared to the rest of the sites. The highest mean NH_4_^+^‐N concentrations were observed at the cropland sites (5.9 mg L^−1^ ± 1.4) and lowest at the river/HLC sites (0.11 ± 0.17). Pairwise comparisons showed (marginally) significantly higher NH_4_^+^‐N at rewetted extraction sites compared to the river/HLCs (*p* = 0.04). A large proportion of samples (55%) had NO_2_^−^‐N concentrations below the Lower Limit of Detection (LLD) of 0.03 mg L^−1^. Both NO_2_^−^‐N (bog: 0.05 ± 0.07, fen: 0.13 ± 0.13) and NH_4_^+^‐N (bog: 1.3 ± 4.0, fen: 1.2 ± 4.5) concentrations were significantly higher in bogs than fens, with no effect of peatland type on NO_3_^−^‐N (*p* = 0.14; Table S2).

### 3.3 Phosphorous

Land use did not have a significant effect on PO_4_^3−^‐P (*p* = 0.14) or TP (*p* = 0.29; Table 1, Figure 2E,F). When examining individual sites, a conventional cropland site on bog peat had notably high concentrations (max = 15.7 mg L^−1^ PO_4_^3−^‐P, 20 mg L^−1^ TP) while the rest of the sites were consistently below ~3 mg L^−1^ (PO_4_^3−^‐P) and ~4 mg L^−1^ (TP). PO_4_^3−^‐P (bog: 1.2 ± 2.2 mg L^−1^, fen: 0.30 ± 1.1 mg L^−1^) and TP (bog: 1.3 ± 2.8 mg L^−1^, fen: 0.1 ± 0.55 mg L^−1^) concentrations were significantly higher at the bog than fen sites (Table S2).

### 3.4 C:N:P Ratios

**FIGURE 3:**
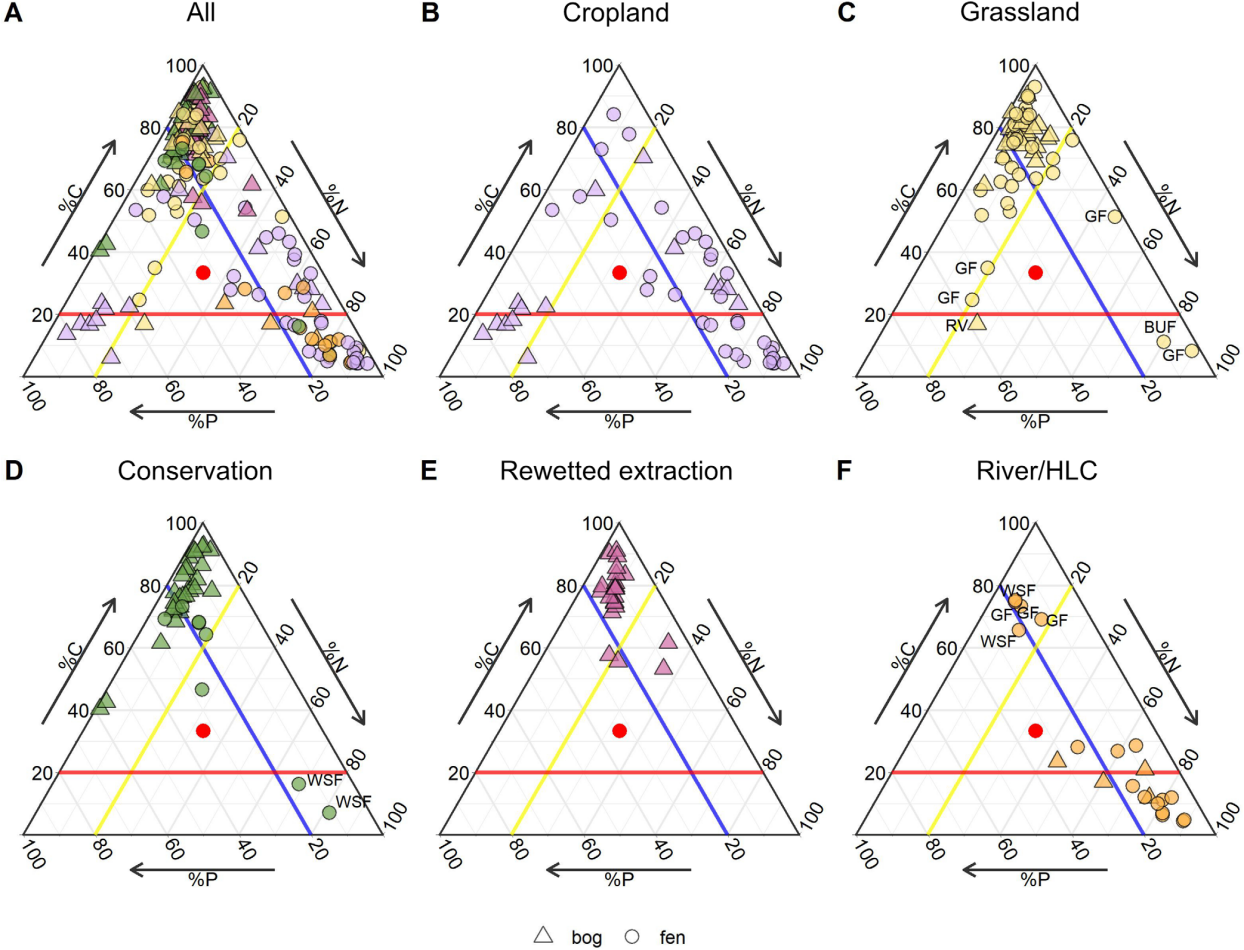
Ternary plots showing the relationships between C, N, and P for all land uses together (A) and separately (B–F). The red point at the centre of the plot marks the Redfield ratio (C:N:P 106:16:1) as a reference point. The coloured lines demark where C (red), N (yellow), and P (blue) are depleted (< 20%) as defined by Smith et al. (2017). Site names labels are shown for points that fall clearly outside the dominant cluster.

The C:N:P ternary plots showed distinct clustering of most land use types (Figure 3A). Croplands did not display a distinct clustering, although the majority of sites were potentially P‐limited (Figure 3B). Grassland sites tended to be N and P co‐limited (Figure 3B). Likewise, conservation‐managed sites also tended to be N and P co‐limited, with two Wicken Sedge Fen sites outlying as C and P co‐limited (Figure 3D). Rewetted extraction sites had a tight clustering in the N and P co‐limitation region (Figure 3E). River/HLC sites tended to be C and P co‐limited, with a small cluster of sites (Wicken Sedge Fen and Great Fen) P and N co‐limited (Figure 3F).

### 3.5 Heavy Metals

Many of the water samples analysed had heavy metal concentrations below the LLD: Pb (86% of samples ≤ LLD), Hg (86%), As (85%), Cr (84%), Zn (45%), Cd (42%), Ni (29%), Cu (18%), Fe (16%), and Mn (15%). There were not enough Hg data points to conduct statistical analysis. Land use had a significant effect on Fe (*p* = 0.04), Mn (*p* = 0.03), and Ni (*p* = 0.001), but not on As (*p* = 0.99), Cd (*p* = 0.62), Cr (*p* = 0.50), Cu (*p* = 0.97), Pb (*p* = 0.40), or Zn (*p* = 0.14). Pairwise comparisons showed significantly higher Fe (*p* = 0.04) and Mn (*p* = 0.03) at rewetted extraction sites (all bog located in north‐west England) compared to the river/HLCs. For Ni, pairwise comparisons showed significantly higher concentrations at cropland sites compared to conservation, grassland and river/HLC sites. The highest heavy metal concentrations were notably clustered near Manchester (all metals) and in eastern England (all metals except Pb and Hg) (Figure S2). Nearly all the metals were positively correlated (Figure S3).

### 3.6 Other Anions and Cations (Al, ca, cl^−^, F^−^, K, Li, Mg, Na, Si, SO_4_^2^ ^−^)

Land use had a significant effect on Ca (*p* = 0.003), Mg (*p* = 0.003), and SO_4_^2−^‐S (*p* < 0.001) but not Al (*p* = 0.15), Cl^−^ (*p* = 0.21), F^−^ (*p* = 0.07), K (*p* = 0.14), Na (*p* = 0.25), Li (*p* = 0.61), and Si (*p* = 0.36) (Figure S4). Ion concentrations were significantly higher in fens than bogs for Al (*p* < 0.0001), Ca (*p* < 0.0001), Cl^−^ (*p* < 0.0001), Mg (*p* = 0.0002), Na (*p* < 0.0001), and SO_4_^2−^‐S (*p* < 0.0001), but no significant differences between peatland type for F^−^ (*p* = 0.12), K (*p* = 0.29), Li (*p* = 0.63), and Si (*p* = 0.06) (Table S2). Similarly, both pH (*p* < 0.0001) and EC (*p* < 0.0001) were respectively 1.3 and three times higher in fens than bogs (Table S2).

### 3.7 Greenhouse Gases

**FIGURE 4:**
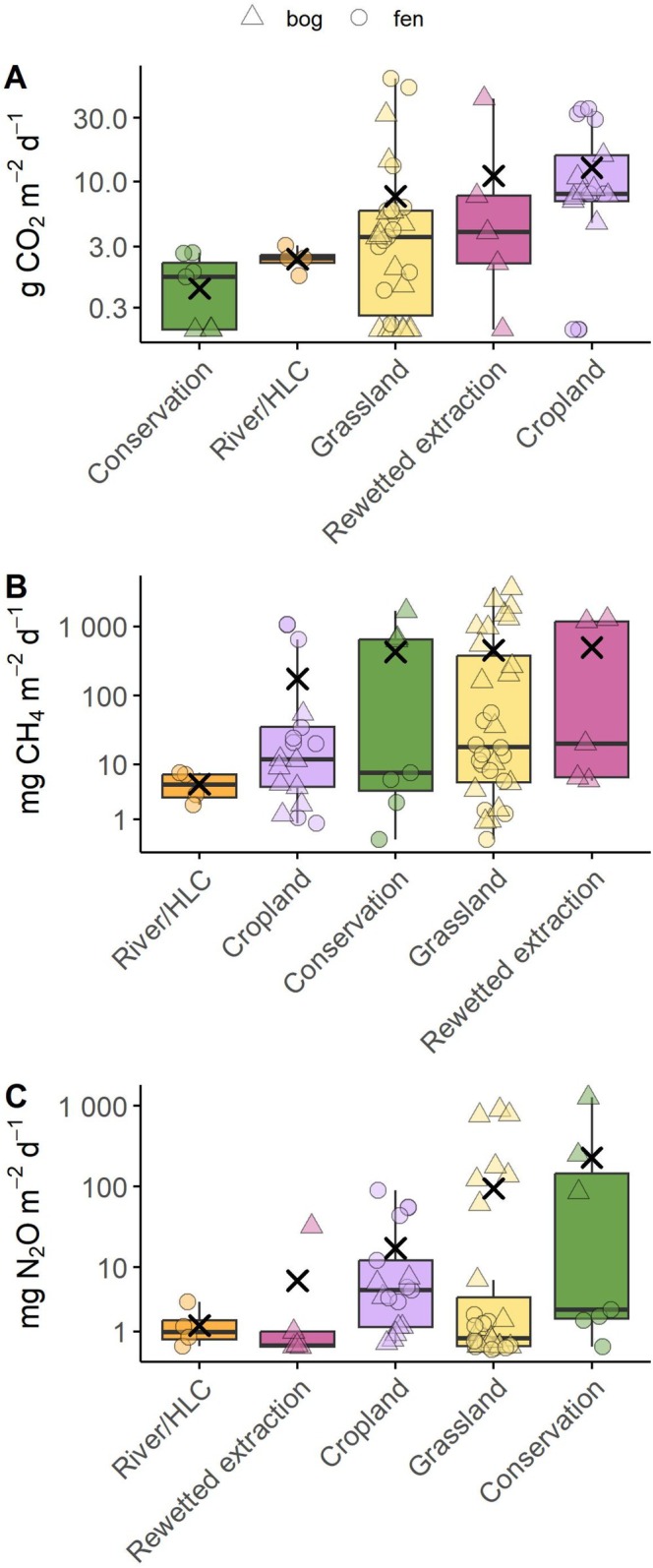
Greenhouse gas (CO_2_, CH_4_, and N_2_O) emissions by land use type, ordered by increasing mean for each gas. Note the variables are plotted on a logarithmic scale for clarity. Box plots display the median (horizontal line), mean (“X”), 25th and 75th percentiles, and whiskers display values 1.5× interquartile range.

Surface waters were on average over‐saturated with GHGs relative to the atmosphere (29.10 ± 42.50 mg L^−1^ CO_2_, 1.27 ± 2.46 mg L^−1^ CH_4_, 0.23 ± 0.71 mg L^−1^ N_2_O), making them net sources of GHG emissions to the atmosphere during the 2024 growing season (8.24 ± 13.10 g CO_2_ m^−2^ d^−1^, 355 ± 696 mg CH_4_ m^−2^ d^−1^, 76.4 ± 230 mg N_2_O m^−2^ d^−1^). We recognise that by only measuring in the daytime, our daily CO_2_ fluxes are likely underestimated, as has been previously demonstrated for streams (Gómez‐Gener et al. 2021). Uptake was observed on 24%, 3%, and 2% of occasions for CO_2_, CH_4_, and N_2_O respectively. Land use did not have a significant effect on GHG concentrations nor emissions (Table S3; Figure 4). None of the GHG concentrations significantly covaried with any of the measured determinands (Figure S5).

## 4 Discussion

Land use had a significant effect on certain water quality parameters, notably high NO_3_^−^ and NH_4_^+^ concentrations at cropland sites. A closer look at nutrient stoichiometry revealed similar patterns at grassland, conservation‐managed, and rewetted extraction sites, indicative of potential N and P co‐limitation, suggesting that rewetting supports a nutrient balance comparable to more natural conditions. Peatland type appeared more important than land use in explaining patterns in most cation and anion concentrations, while site and regional history seemed more important than land use for explaining patterns of heavy metal concentrations.

### 4.1 Key Macronutrient Concentrations

Land use had a significant effect on some macronutrient concentrations (NO_3_^−^, NH_4_^+^), but not others (NO_2_^−^, PO_4_^3−^). The high NO_3_^−^ and NH_4_^+^ concentrations in surface waters draining cropland sites are likely a function of direct fertiliser application and abstraction of river water into ditches, as well as enhanced decomposition of organic matter and resulting N mineralisation (Tiemeyer et al. 2007; van der Laan et al. 2024). Rivers/HLCs also had high NO_3_^−^ and NO_2_^−^ concentrations, likely reflecting the dominance of agricultural land use in their catchments. There were occasional elevated NO_3_^−^‐N concentrations (~5 mg L^−1^) in the conservation‐managed Wicken Sedge Fen, where river water is abstracted onto site to keep water levels high (Peacock et al. 2017). NO_3_^−^ concentrations in rewetted extraction sites were frequently below the LLD (0.2 mg L^−1^ NO_3_^−^ −N), suggesting that rewetting of raised bogs can reverse legacies of high nutrient loads, for example, by retaining nutrients in reduced forms (including as organic compounds) within the anaerobic peat, rather than leaching them in oxidised form as NO_3_^−^ and PO_4_^3−^. The significantly higher NH_4_^+^ at rewetted extraction than River/HLC sites aligns with other studies that have observed an increase in NH_4_^+^ concentrations following rewetting, sourced from the upper, highly decomposed peat layer (van Dijk et al. 2004; Zak and Gelbrecht 2007). The large spread of NH_4_^+^ concentrations at grassland and cropland sites likely obscured any significant effect of land use for these categories. This variability is likely due to the range of management conditions within these land uses. For example, grassland sites can range from extensive systems with low livestock stocking densities and minimal nutrient inputs (which was the case at the majority of our sites) to intensive systems with high stocking densities and substantial nutrient additions.

DOC concentrations were significantly lower in river/HLC sites compared to grassland and conservation‐managed sites. These low DOC concentrations in the river/HLCs highlights how these watercourses are largely disconnected from peatlands. We would expect DOC concentrations to be highest at the drained sites, where aerobic decomposition promotes lateral carbon inputs to ditches (Evans, Renou‐Wilson, and Strack 2016), which was evidenced by high DOC at drained grassland sites. However, high DOC concentrations (max: 157 mg L^−1^) also occurred in the conservation‐managed sites. To some extent this was driven by two sites: Alvanley Lake (Delamere Forest, Cheshire) and the Mini Moss at Risley Moss (Manchester Mosses). High DOC at Alvanley Lake is conceivably due to the fact that this site features extensive flooding, seemingly of considerable depth, and dead trees have been left standing in the water (Cheshire Wildlife Trust 2018), providing a source of organic matter as they decompose. The Mini Moss at Risley has only recently been rewetted fully (last several years) and thus here it may be that a flush of organic matter out of the newly rewetted peat is still ongoing (Herzsprung et al. 2017; Waddington et al. 2008), which may subside in the coming years (Evans, Renou‐Wilson, and Strack 2016; Lundin et al. 2017). The high average DOC concentration at the conservation‐managed sites was also largely skewed by high concentrations at the bog sites, which had DOC concentrations two times higher than fens across the dataset. In contrast, the drained cropland and grassland sites had a greater proportion of fens (70%). These findings highlight the importance of considering site‐specific characteristics—such as peatland type and time since rewetting—in addition to broader land use categories, and their effects on surface water quality.

Contrary to patterns in NO_3_^−^ and NH_4_^+^, rivers/HLCs did not display particularly high mean concentrations of PO_4_^3−^ or P. This is likely because P is retained in soils or sediments before leaching into rivers, or is removed by in‐stream processes once within the fluvial environment (Haygarth and Jarvis 1999; Heathwaite et al. 2000). It is also possible that the river/HLCs receive wastewater effluent which has been subject to P‐removal (Bowes et al. 2010), and/or there is a dilution effect in the river/HLCs that is weaker in the other surface waters.

### 4.2 Nutrient Stoichiometry

Nutrient stoichiometry may be more effective than examining individual nutrient concentrations in describing meaningful ecosystem‐scale patterns in surface waters (Stutter et al. 2018). Croplands showed the most variable nutrient stoichiometry, with no distinct clustering. This is likely due to variable nutrient inputs—both in time and in composition. Grassland, conservation‐managed, and rewetted sites all generally displayed similar clustering indicative of N and P co‐limitation. This suggests relatively balanced but low nutrient availability, characteristic of natural peatland ecosystems (Bragazza et al. 2012). These patterns are a promising indication that rewetting promotes a return to more natural stoichiometric conditions. P and N co‐limitation is also typical of UK headwater streams (Jarvie et al. 2018).

Compared to the other land uses, river/HLC sites had a distinct stoichiometric fingerprint characterized by C and P co‐limitation. This stoichiometry reflects their landscape position, as river/HLC sites are likely more disconnected from peat (lower C inputs), and they receive nutrient‐enriched runoff from their often agriculture‐dominated mineral soil catchments (high N inputs) (Jarvie et al. 2018). Similar results were observed in a national‐scale assessment of UK rivers, where rivers were more P‐limited than headwater streams, attributed to a cumulative anthropogenic nutrient enrichment with increasing catchment scale (Jarvie et al. 2018). Thus, care must be taken if drained peatlands are rewetted (and kept wet) with river/HLC water (as is frequently the case for lowland fens), as its high‐N, low‐C content may have unintended effects on the peatland's ecological and biogeochemical functioning (Bubier et al. 2007). In sum, nutrient stoichiometry appears to be an effective method to examine the effects of land use on peatland surface water quality, with our results suggesting that rewetted sites have similar nutrient stoichiometry to conservation‐managed sites.

### 4.3 Other Ion Concentrations

Land use had a significant effect on Ca, Mg, and SO_4_^2−^, and fen type peatlands generally had higher ion concentrations compared to bogs. Peatland type clearly played a key role here, as most ion concentrations (except for F^−^, K, Li, and Si) were lower at the bog sites and higher at the fen sites. This pattern reflects the underlying differences in peat chemistry and hydrology—where bogs are ombrotrophic systems that receive water and nutrients almost exclusively from atmospheric deposition, while fens are minerotrophic and fed by groundwater or surface water higher in dissolved ions and nutrients (Bleuten et al. 2006). This influence of peatland type is also consistent with pH and EC, both of which had the lowest values for bogs. Together, these results suggest that peatland type is more influential than land use in explaining surface water ion concentration trends in UK lowland peatlands.

### 4.4 Heavy Metal Concentrations

Many heavy metal samples were below the LLD, indicating that waters draining lowland peatlands generally have low concentrations of these potentially toxic elements. However, some of our maximum measured heavy metal concentration values (Cd: 25 μg L^−1^, Pb: 88 μg L^−1^, Ni: 83 μg L^−1^) exceeded the Water Framework Directive environmental water quality standards maximum allowable concentrations for inland surface waters (Cd: 0.45 μg L^−1^, Pb: 14 μg L^−1^, Ni: 34 μg L^−1^) (WFD 2015). When heavy metal concentrations were > LLD, spatial visualization revealed notable clustering in north‐west England at the Manchester Mosses, and a secondary (generally lower magnitude) cluster in eastern England. This notable spatial clustering of high heavy metal concentrations at the Manchester Mosses is likely due to a history of industrial activity in the area. The city of Manchester is ~12 km away and has been an active area for coal mining, chemical production and other intensive industries (Osborne et al. 2024). Indeed, atmospheric Hg emissions continue to be detected in the Manchester area (Lee et al. 2000). Other historic sources for heavy metals in the Manchester Mosses include a nearby World War II munitions factory (Thomas 2016) and inputs of sewage sludge and urban wastes, which deposited directly onto the Manchester Mosses during the 19th century via the railway (Breward 2003). Given the lack of a similar industrial site history at the eastern England sites, we postulate that the measurable heavy metal concentrations there (except for Pb and Hg) are more likely due to the long history of intensive agricultural activity in the East Anglian Fens (Rotherham 2013) and the associated inputs (e.g., manure, fertiliser, irrigation water, sewage sludge and other inputs) (Nicholson et al. 2006). Land use did not have a significant effect on most metals; however, Ni was higher at cropland sites compared to conservation and grassland sites. This pattern may indicate fertilizer origins, as Ni is sometimes even intentionally added to agricultural soils to address Ni deficiency and improve soil fertility (Rabinovich et al. 2024). Across sites, the majority of heavy metal concentrations were significantly positively correlated. Therefore, >LLD values of a single heavy metal (e.g., Zn or Pb) may be used as a proxy to infer high overall heavy metal concentrations in lowland peat surface waters. Site history, notably of industrial and agricultural activities in the wider landscape, should therefore be an important consideration for rewetting of UK lowland peatlands.

### 4.5 Greenhouse Gases

Surface water GHG concentrations and emissions did not differ significantly between land use types and our fluxes were generally similar or higher than means reported for other temperate peatland systems. Our average CO_2_ flux (8 g CO_2_ m^−2^ d^−1^) exceeded fluxes measured in ditches at peat extraction sites in Canada (0.7 g CO_2_ m^−2^ d^−1^ and 5 g CO_2_ m^−2^ d^−1^) (Clark et al. 2023; Strack and Zuback 2013) and UK cropland fen ditches (4 g CO_2_ m^−2^ d^−1^) (Peacock et al. 2017), and was similar to fluxes from agricultural ditches in the Netherlands (9 g CO_2_ m^−2^ d^−1^) (Hendriks et al. 2024). Our average CH_4_ flux (355 mg CH_4_ m^−2^ d^−1^) exceeded fluxes from a drained blanked bog in Wales (14 mg CH_4_ m^−2^ d^−1^) (Green et al. 2018), fen cropland ditches in eastern England, UK (48 mg CH_4_ m^−2^ d^−1^) (Peacock et al. 2017), and pasture‐draining ditches in the Netherlands (206 mg CH_4_ m^−2^ d^−1^) but was less than pasture‐draining ditches in California, USA (622 mg CH_4_ m^−2^ d^−1^) (Teh et al. 2011). Our average N_2_O flux (76.4 mg N_2_O m^−2^ d^−1^) was generally higher than reported values for temperate peatland surface waters including German *Sphagnum* paludiculture ditches (1 mg N_2_O m^−2^ d^−1^) (Daun et al. 2023), pasture‐draining ditches in California, USA (4 mg N_2_O m^−2^ d^−1^) (Teh et al. 2011), and agricultural ditches in Norfolk, UK (13 mg N_2_O m^−2^ d^−1^) (Outram and Hiscock 2012). Our relatively high N_2_O fluxes may be due to the high proportion of intensively‐managed (cropland, grassland) sites compared to conservation‐managed sites in our dataset, the former generally being associated with higher nutrient inputs and therefore higher GHG emissions (Peacock et al. 2021). We also sampled mostly during the growing season, where water temperatures are highest, and microbial activity and therefore GHG emissions tend to peak (Yvon‐Durocher et al. 2014).

We found no significant effect of land use on GHG concentrations nor emissions, likely due to the small sample size in some of the land use categories (e.g., rewetted extraction, *n* = 5). As such, our interpretation of the data trends should be viewed with caution. In drained systems, like croplands and grasslands, we would expect higher rates of aerobic decomposition in the peat, leading to high DOC and CO_2_ fluxes into the ditches (Evans, Renou‐Wilson, and Strack 2016). This may explain the high CO_2_ concentrations we observed at most of the cropland sites—save for three instances of CO_2_ uptake at the Weald Moors site. In addition, nutrients from fertiliser application can enhance microbial activity (Zhang et al. 2024), further contributing to enhanced aquatic CO_2_ production at the cropland sites. Although not observed here, relationships between macronutrient stoichiometry and GHGs have been found in other constructed waterbodies (Åhlén et al. 2023), suggesting that researchers should look beyond individual nutrients in studies of GHGs as well as those of water quality. Together, our results highlight the complex and interactive controls on GHG emissions from lowland peatland surface waters, suggesting that land use alone does not explain the observed patterns.

### 4.6 Conclusions and Implications

Here, we investigated land use, peatland type, and site/regional history related trends in UK lowland peat surface water quality. There were no consistent negative impacts of rewetting former peat extraction sites on nutrient stoichiometry, water quality, or GHGs, which is an important finding given the large‐scale peatland restoration and rewetting activities planned around the world, for example, in England (Department for Environment, Food & Rural Affairs [DEFRA] 2021), Europe (Regulation (EU) 2024/1991), and Indonesia (Lestari et al. 2024). Site and regional history were important for heavy metal concentrations, which occasionally exceeded water quality standards. As such, industrial and agricultural activities (historic and current) at the site and in the wider landscape, should be an important consideration when rewetting peatlands. Overall, these results provide a valuable foundation for examining the effects of land use on surface water quality in UK lowland peatlands that can be used to guide global peatland management and restoration strategies that promote both climate mitigation and water quality protection.

## Author Contributions

**Teresa Silverthorn:** formal analysis, visualization, writing – original draft, writing – review and editing. **Mike Peacock:** conceptualization, funding acquisition, investigation, writing – original draft, writing – review and editing. **Francesca Baker:** investigation, writing – review and editing. **Chris D. Evans:** funding acquisition, writing – review and editing. **Caroline Gurd:** investigation, writing – review and editing. **Josh Oakley:** investigation, writing – review and editing. **Ross Morrison:** funding acquisition, writing – review and editing. **Ian Holman:** funding acquisition, writing – review and editing. **Angus McEwen:** investigation, writing – review and editing. **Chris Bell:** writing – review and editing, funding acquisition. **Graham Howell:** investigation, writing – review and editing. **Dan Aberg:** investigation, writing – review and editing. **Francesca Southon:** investigation, writing – review and editing.

## Funding

This work was supported by the Environment Agency and Svenska Forskningsrådet Formas, 2020‐00950.

## Conflicts of Interest

The authors declare no conflicts of interest.

## Supporting information


**Table S1:** Site names and their attributes (site name, site code, minimum number of unique sampling points [*n*], land use, peatland type, number of sampling visits, and coordinates) organized by region. Note that each site often had several sampling points, therefore the coordinates are indicative of one of these points.
**Table S2:** Type III ANOVA results from LMMs assessing peatland type (bog vs. fen) on water quality variables. For each variable the degrees of freedom (numerator and denominator), *F* value, *p* value, transformation (logarithmic, square root, cube root), and number of outliers are noted. Significant effects are bolded.
**Table S3:** ANOVA results from LMs assessing land use effects on greenhouse gas concentrations and fluxes. For each variable the degrees of freedom, sum of squares, *F* value, *p* value, and type of transformation.
**Table S4:** Lower limit of detection (LLD) and limit of quantification (LoQ) values for determinands analysed (and their method of analysis in brief).
**Figure S1:** Temporal dynamics of key nutrient concentrations in lowland peat surface waters by land use type. Note that the lines connecting the means (± SE) are to aid in visual interpretation and do not indicate continuous measurements. Note that DOC was not measured on November samples.
**Figure S2:** Spatial distribution of dissolved heavy metal concentrations across study sites. Circle sizes represent concentrations, with only concentration values > LLD plotted.
**Figure S3:** Spearman correlation matrix of water quality data. To account for multiple comparisons in the correlation matrix, *p*‐values were adjusted using a Bonferroni correction. The colour scale indicates the strength and direction of correlations: blue for positive and red for negative. Insignificant correlations are left blank.
**Figure S4:** Anion and cation concentrations in lowland peat surface waters by land use type, ordered by increasing mean for each determinand. Note that some variables are plotted on a logarithmic scale for clarity. Box plots display the median (horizontal line), mean (“X”), 25th and 75th percentiles, and whiskers display values 1.5× interquartile range. Bars sharing the same lowercase letters (or with no letters) are not significantly different (pairwise Tukey adjusted, *p* < 0.05).
**Figure S5:** Spearman correlation matrix of greenhouse gas concentrations and a subset of associated water quality data. To account for multiple comparisons in the correlation matrix, *p*‐values were adjusted using a Bonferroni correction. The colour scale indicates the strength and direction of correlations: blue for positive and red for negative. Insignificant correlations are left blank (*p* < 0.05).
**Text S1:** Laboratory analyses.

## Data Availability

The data that support the findings of this study are openly available in Figshare at 10.6084/m9.figshare.30740297. The programming code to replicate analyses is available in Github at https://github.com/TeresaSilverthorn/Lowlandpeat.
