# Supplementary material for: Land‐Use Effects on Surface Water Quality in Temperate Lowland Peatlands
Source: Glob Chang Biol. 2026 Jul 5;32(7):e70988. doi: 10.1111/gcb.70988 (PMC13334241; doi:10.1111/gcb.70988)
Supplement: Supplementary file 1 — Table S1: Site names and their attributes (site name, site code, minimum number of unique sampling points [n], land use, peatland type, number of sampling visits, and coordinates) organized by region. Note that each site often had several sampling points, therefore the coordinates are indicative of one of these points. Table S2: Type III ANOVA results from LMMs assessing peatland type (bog vs. fen) on water quality variables. For each variable the degrees of freedom (numerator and denominator), F value, p value, transformation (logarithmic, square root, cube root), and number of outliers are noted. Significant effects are bolded. Table S3: ANOVA results from LMs assessing land use effects on greenhouse gas concentrations and fluxes. For each variable the degrees of freedom, sum of squares, F value, p value, and type of transformation. Table S4: Lower limit of detection (LLD) and limit of quantification (LoQ) values for determinands analysed (and their method of analysis in brief). Figure S1: Temporal dynamics of key nutrient concentrations in lowland peat surface waters by land use type. Note that the lines connecting the means (± SE) are to aid in visual interpretation and do not indicate continuous measurements. Note that DOC was not measured on November samples. Figure S2: Spatial distribution of dissolved heavy metal concentrations across study sites. Circle sizes represent concentrations, with only concentration values > LLD plotted. Figure S3: Spearman correlation matrix of water quality data. To account for multiple comparisons in the correlation matrix, p‐values were adjusted using a Bonferroni correction. The colour scale indicates the strength and direction of correlations: blue for positive and red for negative. Insignificant correlations are left blank. Figure S4: Anion and cation concentrations in lowland peat surface waters by land use type, ordered by increasing mean for each determinand. Note that some variables are plotted on a logarithmic scale for [file GCB-32-e70988-s001.docx]

## Supplementary Materials for

# Land-use effects on surface water quality in temperate lowland peatlands

Teresa Silverthorn^1,2*^, Dan Aberg^3^, Francesca Baker^1^, Chris Bell^4^, Chris D. Evans^4^, Caroline Gurd^5^, Ian Holman^6^, Graham Howell^5^, Angus McEwen^5^, Ross Morrison^7^, Josh Oakley^5^, Francesca Southon^1^, Mike Peacock^1,8*^

**Affiliations**

1. Department of Geography and Planning, School of Environmental Sciences, University of Liverpool, Liverpool, UK
2. School of Environmental Science, Simon Fraser University, Burnaby, Canada
3. School of Environmental and Natural Sciences, Bangor University, Bangor, UK
4. UK Centre for Ecology & Hydrology, Bangor, United Kingdom
5. School of Environment, Earth and Ecosystem Sciences, The Open University, Milton Keynes, UK
6. Cranfield Water Science Institute, Cranfield University, Cranfield, UK
7. UK Centre for Ecology & Hydrology, Wallingford, United Kingdom
8. Department of Aquatic Sciences and Assessment, Swedish University of Agricultural Sciences, Uppsala, Sweden

* Corresponding authors: Teresa Silverthorn (teresa_silverthorn@sfu.ca, teresa.silverthorn@gmail.com) and Mike Peacock (m.peacock@liverpool.ac.uk)

| **Table S1** Site names and their attributes (site name, site code, minimum number of unique sampling points [*n*], land use, peatland type, number of sampling visits, and coordinates) organized by region. Note that each site often had several sampling points, therefore the coordinates are indicative of one of these points. | | | | | | |
| --- | --- | --- | --- | --- | --- | --- |
| Site name | Site code | *n* | Land use | Peatland type | Sampling visits | Lat, Lon |
| **Manchester Mosses (north-west England)** | | | | | | |
| Foresters | FF | 3 | Grassland | Bog | 4 | 53.45394, -2.453528 |
| Holcroft Moss | HOL | 1 | Conservation-managed | Bog | 3 | 53.43633, -2.474194 |
| Little Woolden Moss | LWM | 4 | Rewetted extraction | Bog | 3 | 53.44769, -2.468556 |
| Moss Side Farm | MS | 2 | Grassland | Bog | 1 | 53.47025, -2.44383 |
| Railway View | RV | 3 | Grassland | Bog | 4 | 53.46258, -2.444667 |
| Rindle | RIN | 1 | Cropland | Bog | 1 | 53.48242, - 2.447778 |
| Risley Main | RIS-MAI | 1 | Rewetted extraction | Bog | 3 | 53.42356, - 2.500361 |
| Risley Mini | RIS-MIN | 1 | Conservation-managed | Bog | 3 | 53.42356, -2.500361 |
| Stuarts | STU | 2 | Grassland | Bog | 1 | 53.46192, -2.428111 |
| **Cheshire (north-west England)** | | | | | | |
| Delamere Forest | DEL | 2 | Conservation managed | Bog | 4 | 53.23681, -2.681472 |
| **Lancashire Mosses (north-west England)** | | | | | | |
| Cheshire Lines | CL | 2 | Cropland, River/HLC | Fen | 4 | 53.567528, -3.013722 |
| Holiday Moss | HOM | 1 | Conservation-managed | Bog | 2 | 53.51119, - 2.770472 |
| Leighton Moss | LEI | 1 | Conservation-managed | Fen | 1 | 54.16769, - 2.791917 |
| Meadow Lane | ML | 2 | Cropland, River/HLC | Bog | 5 | 53°36'49.3"N 2°48'37.9"W |
| Rufford Boundary Sluice | RBS | 2 | Cropland | Fen | 5 | 53.632722, -2.855111 |
| Winmarleigh Carbon Farm | WCF | 1 | Rewetted extraction | Bog | 1 | 52.30592, - 2.849889 |
| Wright’s Farm | WF | 2 | Cropland | Bog | 5 | 53.68167, -2.877806 |
| **East Anglia (eastern England)** | | | | | | |
| Burwell Fen | BUF | 4 | Grassland, River/HLC | Fen | 4 | 52.29911, 0.269861 |
| Great Fen | GF | 7 | Grassland, River/HLC | Fen | 5 | 52.49806, -0.229889 |
| Holme Fen | HF | 3 | Conservation-managed | Bog | 4 | 52.49014, -0.231444 |
| Little Common Farm | LC | 1 | Cropland | Fen | 3 | 52.44111, -0.245056 |
| Rosedene Farm | RG | 4 | Cropland | Fen | 4 | 52.52208, 0.475639 |
| Roughs Farm | SW | 5 | Cropland, River/HLC | Fen | 4 | 52.43711, -0.258722 |
| Tubney Fen | TUF | 2 | Grassland | Fen | 4 | 52.27644, 0.278611 |
| Wicken Baker’s Fen | WBF | 2 | Grassland | Fen | 4 | 52.30592, 0.292500 |
| Wicken Sedge Fen | WSF | 5 | Conservation-managed, River/HLC | Fen | 4 | 52.31086, 0.290472 |
| Woodwalton Fen | WW | 2 | Conservation-managed fen | Fen | 4 | 52.44886, -0.190639 |
| **Wales** | | | | | | |
| Cors Fochno | CFO | 2 | Conservation-managed | Bog | 1 | 52.50772, -4.012806 |
| **Cumbria Mosses (north-west England)** | | | | | | |
| Foulshaw Moss | FOM | 1 | Conservation-managed | Bog | 1 | 54.24631, -2.833583 |
| **Somerset (south-west England)** | | | | | | |
| Gold Corner Farm | GC | 1 | Grassland | Fen | 1 | 51.17967, -2.888667 |
| West Sedgemoor | WSE | 2 | Grassland, River/HLC | Fen | 1 | 51.02653, -2.913417 |
| **Norfolk Broads (eastern England)** | | | | | | |
| Langley Street | LST | 2 | Grassland | Fen | 1 | 52.56206, 1.499611 |
| **Shopshire** | | | | | | |
| Weald Moors | WM | 3 | Cropland | Fen | 5 | 52.76633, -2.43527 |

| Table S2 Type III ANOVA results from LMMs assessing peatland type (bog vs. fen) on water quality variables. For each variable the degrees of freedom (numerator and denominator), *F* value, *p* value, transformation (logarithmic, square root, cube root), and number of outliers are noted. Significant effects are bolded. | | | | | | |
| --- | --- | --- | --- | --- | --- | --- |
| Variable | *df* (num) | *df* (den) | *F* value | *p* value | transform | outliers |
| pH | **1** | **28.86** | **32.87** | **< 0.0001** | **-** | **-** |
| EC | **1** | **27.51** | **37.07** | **< 0.0001** | **log** | **1** |
| DOC | **1** | **25.96** | **22.28** | **< 0.0001** | **log** | **1** |
| NO_3_^−^-N | 1 | 28.07 | 2.33 | 0.14 | log | - |
| NH_4_^+^-N | 1 | 28.23 | 4.96 | 0.03 | log | - |
| NO_2_^−^-N | **1** | **24.78** | **27.30** | **< 0.0001** | **log** | **-** |
| PO_4_^3−^-P | **1** | **31.52** | **6.32** | **0.02** | **log** | **-** |
| P | **1** | **31.38** | **17.73** | **0.0002** | **log** | **-** |
| As | **1** | **18.08** | **6.06** | **0.02** | **log** | **1** |
| Cd | **1** | **29.21** | **16.10** | **0.0004** | **log** | **1** |
| Cr | 1 | 0.03 | 1.43 | 0.23 | cubrt | 1 |
| Cu | **1** | **29.48** | **7.97** | **0.008** | **log** | **-** |
| Fe | **1** | **33.94** | **48.08** | **< 0.0001** | **log** | **3** |
| Mn | **1** | **27.66** | **18.25** | **0.0002** | **log** | **2** |
| Ni | 1 | 21.61 | 0.40 | 0.53 | log | - |
| Pb | **1** | **24.79** | **9.96** | **0.004** | **log** | **-** |
| Zn | **1** | **31.14** | **16.83** | **0.0003** | **log** | **1** |
| Al | **1** | **30.57** | **49.77** | **< 0.0001** | **log** | **-** |
| Ca | **1** | **30.24** | **36.10** | **< 0.0001** | **log** | **-** |
| Cl^−^ | **1** | **28.47** | **27.78** | **< 0.0001** | **log** | **-** |
| F^−^ | 1 | 31.02 | 2.59 | 0.12 | sqrt | - |
| K | 1 | 27.69 | 1.16 | 0.29 | log | - |
| Li | 1 | 15.50 | 0.24 | 0.63 | log | - |
| Mg | **1** | **29.77** | **17.79** | **0.0002** | **log** | **-** |
| Na | **1** | **29.71** | **31.44** | **< 0.0001** | **log** | **-** |
| Si | 1 | 27.98 | 3.92 | 0.06 | log | - |
| SO_4_^2−^-S | **1** | **29.52** | **24.52** | **< 0.0001** | **log** | **-** |

| Table S3 ANOVA results from LMs assessing land use effects on greenhouse gas concentrations and fluxes. For each variable the degrees of freedom, sum of squares, *F* value, *p* value, and type of transformation. | | | | | |
| --- | --- | --- | --- | --- | --- |
| Variable | *df* | *Sum sq* | *F* value | *p* value | transform |
| *p*CO_2_ | 4 | 85.45 | 1.73 | 0.16 | log |
| *p*CH_4_ | 4 | 29.96 | 1.20 | 0.32 | log |
| *p*N_2_O | 4 | 36.46 | 1.51 | 0.21 | log |
| CO_2_ flux | 4 | 11.90 | 2.40 | 0.06 | log |
| CH_4_ flux | 4 | 20.16 | 0.90 | 0.47 | log |
| N_2_O flux | 4 | 19.15 | 1.20 | 0.32 | log |

| Table S4 Lower Limit of Detection (LLD) and Limit of Quantification (LoQ) values for determinands analysed (and their method of analysis in brief). | | | |
| --- | --- | --- | --- |
| Element | LLD | LLQ | Method |
| TIC mg L^-1^ | 0.60 | 1.79 | Elementar/Shimadzu TOC-L |
| TC mg L^-1^ | 1.26 | 3.58 | Elementar/Shimadzu TOC-L |
| DOC (diff) mg L^-1^ | 1.02 | 2.88 | Elementar/Shimadzu TOC-L |
| NO_3_^−^ mg L^-1^ | 0.87 | 1.61 | IC |
| NH_4_^+^ mg L^-1^ | 0.045 | 0.136 | IC |
| NO_2_^−^ mg L^-1^ | 0.11 | 0.33 | IC |
| PO_4_^3−^ mg L^-1^ | 0.61 | 1.03 | IC |
| P µg L^-1^ | 14.0 | 35.6 | ICP-OES SiSP |
| As µg L^-1^ | 16.4 | 53.1 | ICP-OES trace |
| Cd µg L^-1^ | 0.9 | 2.2 | ICP-OES trace |
| Cr µg L^-1^ | 2.2 | 6.0 | ICP-OES trace |
| Cu µg L^-1^ | 1.2 | 5.3 | ICP-OES trace |
| Fe µg L^-1^ | 14.3 | 42.0 | ICP-OES trace |
| Mn µg L^-1^ | 0.7 | 2.0 | ICP-OES trace |
| Ni µg L^-1^ | 2.7 | 7.7 | ICP-OES trace |
| Pb µg L^-1^ | 8.6 | 25.7 | ICP-OES trace |
| Zn µg L^-1^ | 3.8 | 17.8 | ICP-OES trace |
| Al µg L^-1^ | 18.8 | 60.6 | ICP-OES trace |
| Ca mg L^-1^ | 0.634 | 1.928 | IC |
| Cl^−^ mg L^-1^ | 0.35 | 0.69 | IC |
| F^−^ mg L^-1^ | 0.04 | 0.13 | IC |
| K mg L^-1^ | 0.141 | 0.429 | IC |
| Li mg L^-1^ | 0.044 | 0.135 | IC |
| Mg mg L^-1^ | 0.047 | 0.142 | IC |
| Na mg L^-1^ | 1.781 | 4.657 | IC |
| Si µg L^-1^ | 11.1 | 27.2 | ICP-OES SiSP |
| SO_4_^2−^ mg L^-1^ | 0.08 | 0.23 | IC |

**Supplementary Text 1 – Laboratory analyses**

Note that for approximately 77% of samples PO_4_ > P (when both concentrations were expressed as mg P L^-1^). This artefact generally arises when concentrations are close to detection limits; evidenced by 20% of the PO_4_ > P subset having concentrations less than the limit of quantification (LoQ), and the median difference between PO_4_ and P for the 77% subset was small (0.1 mg L^-1^). In some cases, it may also arise due to slight changes in concentrations due to different storage times.

An initial set of analyses including blanks and reference standards over three batches of samples was used to define lower limits of reportable data, following Clesceri et al. (1998):

- Lower limit of detection (LLD) = 2*1.645* s +mean blank (where s = standard deviation of blanks), and is the point where type I and type 2 errors are both equal at 5% (i.e. 5% likelihood of identifying a false positive of a false negative).
- Limit of quantification (LoQ) = 10*s. Above this level quantification is considered reliable.

For each subsequent batch of samples analysed, blanks were included and used to calculate a Method Detection Limit (MDL). Here, the blanks are simply de-ionised water and the MDL may be lower than LLD. For the IC and TOC analyses, the blanks did not give quantifiable data for blanks for some analytes, preventing statistical analysis. Instead, the lowest quantifiable standard was used. For all batches, data lower than the LLD for each analyte were reported as less than that value (e.g. Cl <0.3 mg L^-1^) and coded as LLD*0.5 in our dataset for analysis and visualization. For batches/analytes where the MDL is higher than this value, data lower than the batch MDL for each analyte are reported as less than that value. See Table S4 for LLD and LoQ values for each determinand. Dilutions were compared to neat wherever possible, with no matrix effects observed. More abundant elements were present at low concentrations and were analysed at lower dilutions, while for a significant proportion of samples there was a need for multiple sequential dilutions to collect the full range of analytes.

In late September and early October 2024, we additionally sampled 60 sites for analysis of dissolved Hg. Sites included field ditches and rivers/HLCs. Sampling and analysis were done according to the UKAS-accredited (ISO/IEC 17025:2017) National Laboratory Service (NLS) protocols. Sampled were filtered in the field using 0.45 µm syringe filters, and injected into 100 ml Pyrex bottles containing 4 ml of 17% hydrochloric acid. Bottles were then transported to the NLS in temperature-controlled boxes for analysis of dissolved Hg via cold vapour atomic fluorescence spectroscopy.


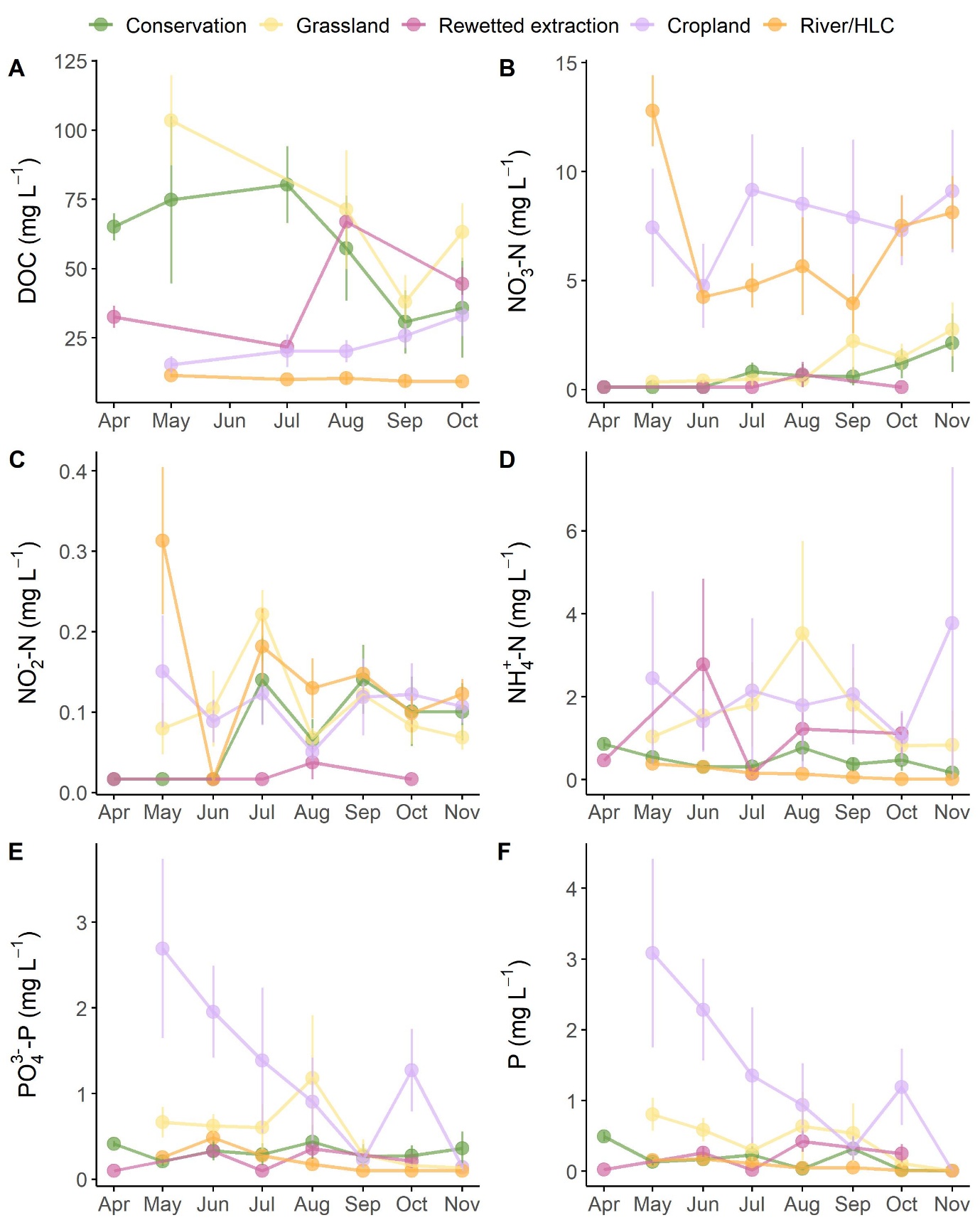


**Figure S1**. Temporal dynamics of key nutrient concentrations in lowland peat surface waters by land use type. Note that the lines connecting the means (± SE) are to aid in visual interpretation and do not indicate continuous measurements. Note that DOC was not measured on November samples.


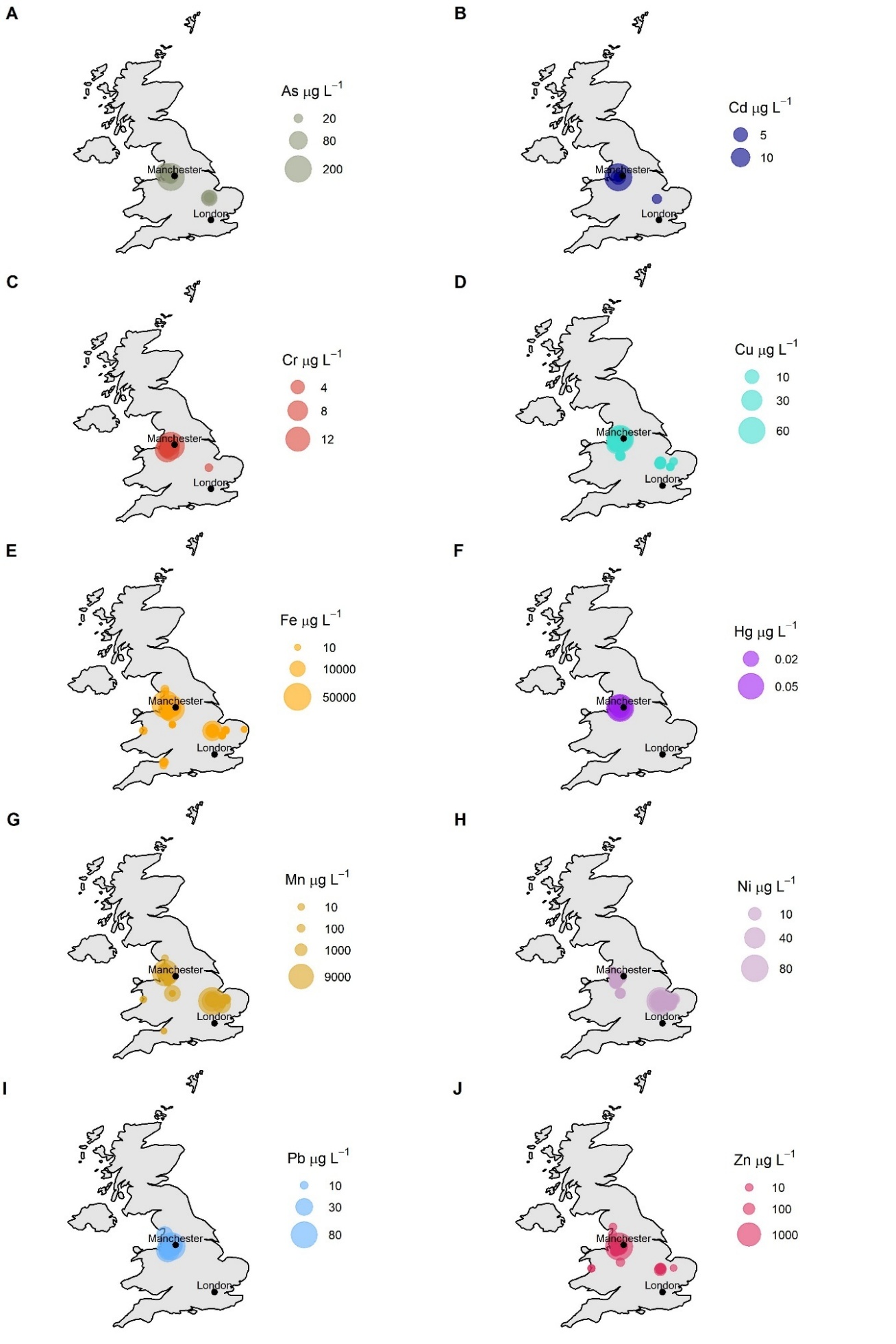
**Figure S2**. Spatial distribution of dissolved heavy metal concentrations across study sites. Circle sizes represent concentrations, with only concentration values >LLD plotted.


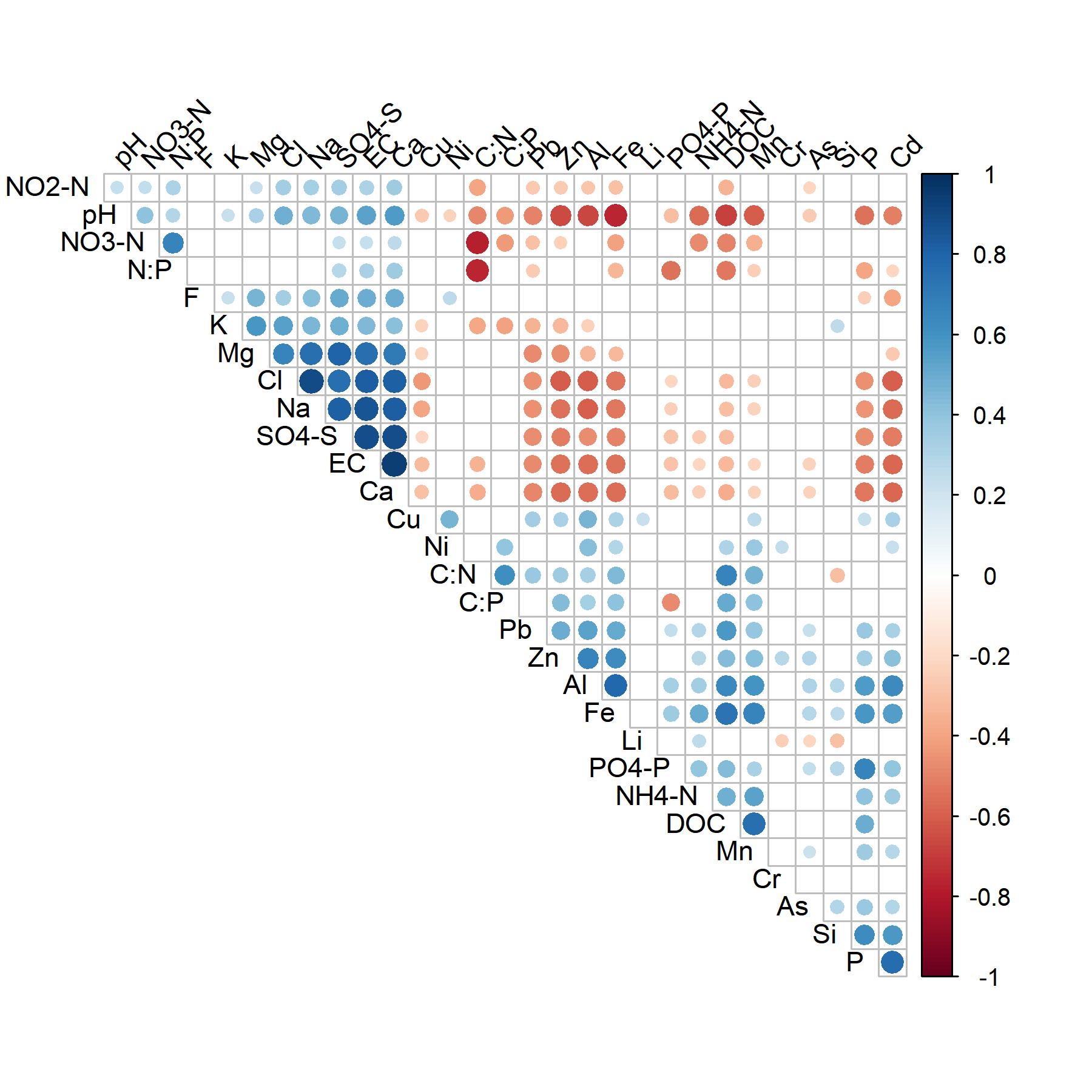
 **Figure S3.** Spearman correlation matrix of water quality data. To account for multiple comparisons in the correlation matrix, *p*-values were adjusted using a Bonferroni correction. The colour scale indicates the strength and direction of correlations: blue for positive and red for negative. Insignificant correlations are left blank.


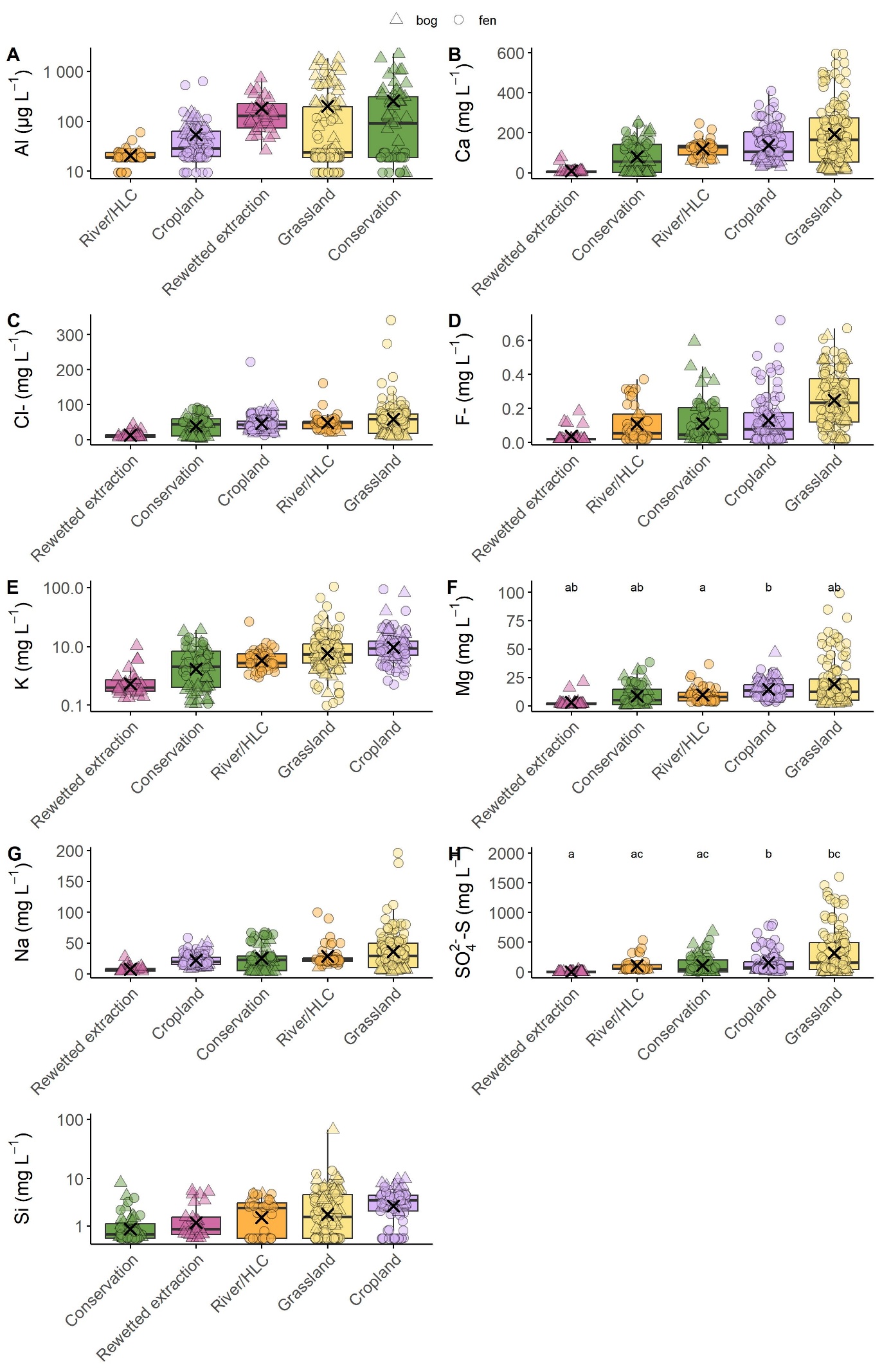
**Figure S4.** Anion and cation concentrations in lowland peat surface waters by land use type, ordered by increasing mean for each determinand. Note that some variables are plotted on a logarithmic scale for clarity. Box plots display the median (horizontal line), mean (“X”), 25^th^ and 75th percentiles, and whiskers display values 1.5x interquartile range. Bars sharing the same lowercase letters (or with no letters) are not significantly different (pairwise Tukey adjusted, *p* < 0.05).


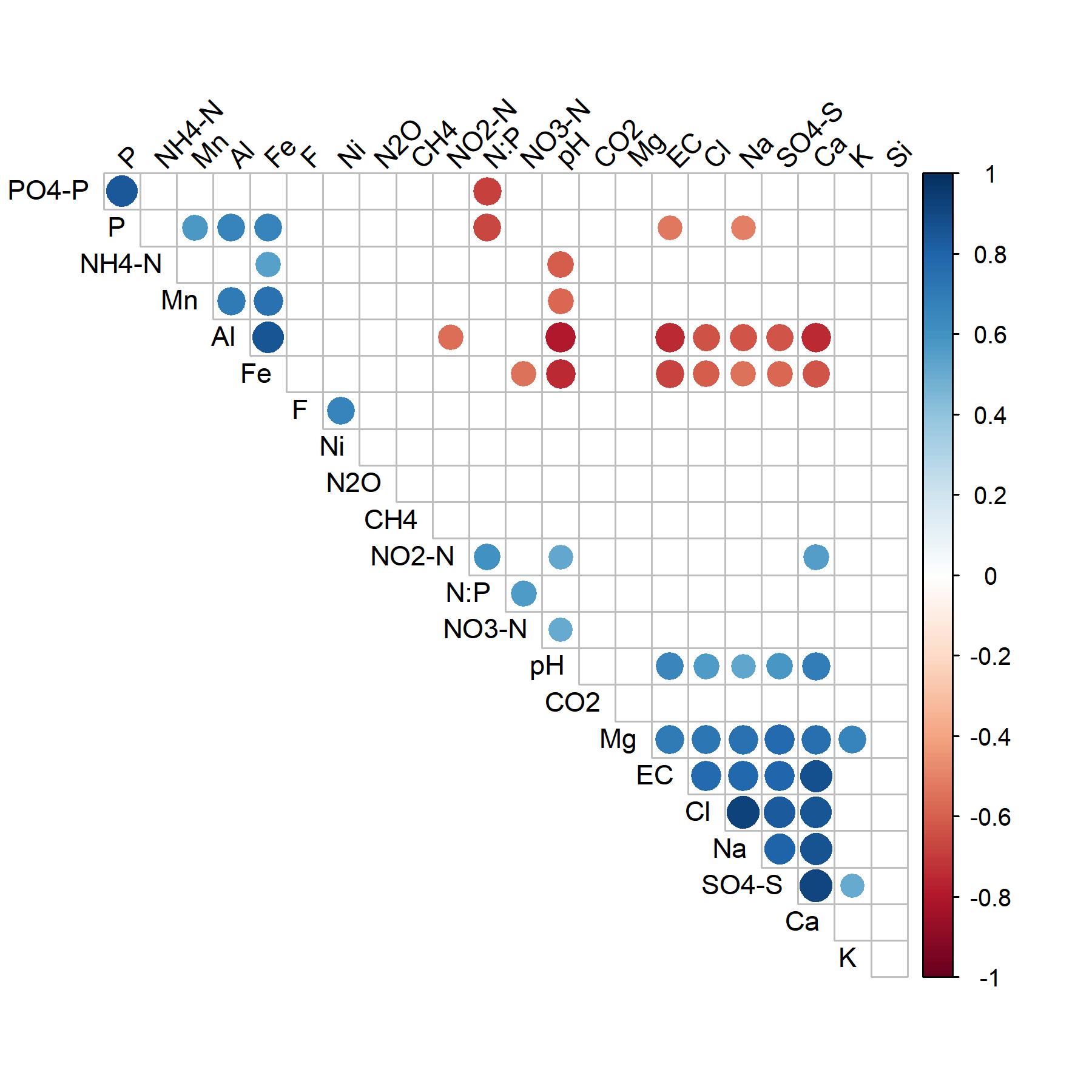


**Figure S5.** Spearman correlation matrix of greenhouse gas concentrations and a subset of associated water quality data. To account for multiple comparisons in the correlation matrix, *p*-values were adjusted using a Bonferroni correction. The colour scale indicates the strength and direction of correlations: blue for positive and red for negative. Insignificant correlations are left blank (*p* < 0.05).
